# Ralstonia pickettii as an emerging pediatric pathogen: a mini-review of current evidence

**DOI:** 10.3389/fped.2026.1763328

**Published:** 2026-02-23

**Authors:** Christian Alberto Rodriguez-Saldaña, Mirtha Chiroque-Zavala, Milagros Quindes-Jaimes, Gianella María Muñoz-Vílchez, Karla Inés Alcántara-Sánchez, Adriana Montoya-Reátegui, Anabella Quiroga-Taboada, Luis Gabriel Farfán-Chávez, Daniel Reyes-Chávez, Juan José Flores-Rodriguez

**Affiliations:** 1 Department of Pediatrics, Peru–Korea Friendship Santa Rosa II-2 Hospital, Piura, Peru; 2 Department of Infectious Diseases, Peru–Korea Friendship Santa Rosa II-2 Hospital, Piura, Peru; 3 Department of Pediatrics, Hospital II Talara, Piura, Peru; 4 Department of Infectious Diseases, José Cayetano Heredia III-1 Hospital, EsSalud, Piura, Peru; 5 Department of Internal Medicine, José Cayetano Heredia III-1 Hospital, EsSalud, Piura, Peru

**Keywords:** bacteremia, bio-film, contaminated solutions, healthcare-associated infection, neonatal intensive care, outbreak investigation, pediatric infections, Ralstonia pickettii

## Abstract

**Background:**

Ralstonia pickettii has gained relevance in pediatric healthcare due to its persistence in water systems, biofilm formation and contamination of medical solutions. This review summarizes current evidence on its epidemiology, environ-mental reservoirs, clinical features, diagnostic limitations and therapeutic considera-tions in children.

**Methods:**

A narrative search was conducted in PubMed, Scopus, Web of Science and Embase for studies published from January 1990 to 30 November 2025. Pediatric cases, outbreak reports, environmental studies with clinical relevance and microbiological reviews were included. Heterogeneity in design and reporting justified a narrative synthesis.

**Results:**

R. pickettii is the predominant Ralstonia species in pedi-atric infections and is closely linked to contaminated aqueous products and respiratory equipment. Neonates and immunocompromised children are most affected, with man-ifestations ranging from colonization to severe sepsis. Diagnostic systems frequently misidentify the organism, and molecular tools improve accuracy when combined with clinical assessment. Susceptibility patterns are variable and influenced by intrinsic re-sistance mechanisms and biofilm. Effective treatment often requires targeted therapy and device removal. Outbreak investigations consistently identify contaminated solu-tions and water systems as primary sources.

**Conclusions:**

R. pickettii is an emerging pathogen in pediatric care. Improving diagnostic accuracy, strengthening environ-mental control and ensuring safe handling of water-based solutions and medical devices are essential to reduce its clinical impact.

## 1 Introduction

*Ralstonia pickettii* was initially described in the 1970s as *Pseudomonas pickettii*, later reclassified within *Burkholderia*, and ultimately assigned to the genus *Ralstonia* in 1995 in recognition of Erwin F. Ralston and M. J. Pickett (1). It is now increasingly recognized as a cause of healthcare-associated infections in pediatric settings, supported by its ability to persist in water systems, form biofilms, and resist commonly used disinfection procedures (2). Multiple outbreaks have shown that this organism can contaminate intravenous solutions, saline flushes, disinfectants, and other products presumed to be sterile, creating an avenue for infection in children who are already vulnerable due to prematurity, chronic illness, or invasive medical support (3–5).

The capacity of *R. pickettii* to thrive in water distribution systems and adhere to the internal surfaces of medical devices presents an additional challenge for pediatric units, where central lines, respiratory equipment, and continuous infusions are widely used (3, 6). Over the past decade, reports have described clusters of bloodstream infections in pediatric intensive care settings, including high-risk populations such as hematopoietic stem cell transplant recipients, emphasizing the organism's clinical relevance in children (7, 8). These episodes often manifest with subtle signs, which can delay recognition, particularly when the isolate is initially misinterpreted as a contaminant rather than a true pathogen (5).

The global distribution of outbreaks indicates that the issue is not confined to high-income healthcare systems. In Latin America, reports of *R. pickettii* infection linked to contaminated intravenous fluids or catheter-associated events illustrate its ability to exploit structural and procedural gaps across diverse clinical environments (9–11). These regional experiences support its position as an emerging pathogen in both adult and pediatric care.

Despite an increasing number of documented cases, current knowledge remains fragmented. Inconsistent diagnostic practices, slow growth in culture, and the enduring perception of *R. pickettii* as a contaminant contribute to underrecognition. These limitations hinder the establishment of standardized approaches for surveillance, diagnosis, and clinical management. Consolidating available evidence is therefore necessary to clarify the epidemiological role of *R. pickettii*, characterize its clinical expression in children, and identify opportunities for improved diagnostic and preventive strategies.

This mini-review synthesizes pediatric and multidisciplinary evidence to provide an updated perspective on the clinical importance of *R. pickettii*, with emphasis on epidemiology, environmental reservoirs, clinical presentation, diagnostic challenges, and therapeutic considerations.

## 2 Materials and methods

This narrative mini-review was developed to summarize clinically relevant information on *Ralstonia pickettii* in pediatric settings. The focus was on environments where children are most vulnerable, including neonatal units, pediatric intensive care and general inpatient wards. A structured search was conducted in PubMed, Scopus, Web of Science and Embase for articles published from January 1990 to 30 November 2025. Earlier publications were included only when they provided essential historical context or described sentinel outbreaks that informed current knowledge. The search strategy combined controlled vocabulary and free-text terms using the expression (“Ralstonia pickettii” OR “Ralstonia species”) AND (“pediatric” OR “children” OR “neonate”), which identified clinical reports, outbreak investigations, microbiological studies and review articles related to pediatric care. The full detailed search strategy for each database is provided in Appendix A.

Study selection followed a concept-driven approach consistent with narrative review methodology. Articles were included when they reported pediatric or neonatal cases attributed to *R. pickettii*, documented outbreaks in pediatric settings, examined environmental sources with clinical relevance or contributed epidemiological or microbiological insights applicable to children. Studies focused solely on adults, centered on other *Ralstonia* species without clear relevance to *R. pickettii*, lacking accessible full texts or consisting only of commentary were excluded. Mixed-population studies were considered only when pediatric data were explicitly identifiable. Screening was completed in two steps. Titles and abstracts were reviewed first, followed by full-text evaluation by two authors who resolved any discrepancies through discussion. Risk-of-bias tools were not applied due to the narrative design, although greater interpretive weight was given to outbreak investigations supported by microbiological confirmation and well-defined pediatric populations.

Extracted information included patient characteristics, clinical findings, diagnostic approaches, antimicrobial susceptibility, suspected contamination sources and outbreak-control measures. The literature consisted mainly of case reports, small series, outbreak descriptions, microbiological studies and environmental analyses. Because of this heterogeneity, a narrative synthesis was selected. This approach allowed the integration of clinical, microbiological and environmental perspectives relevant to *R. pickettii* infections in children.

No new data, materials or software code were generated. All information originated from publicly available sources, and ethical approval was not required. Generative artificial intelligence tools were used only to support linguistic clarity. All decisions regarding content, interpretation and structure were made solely by the authors.

## 3 Results

### 3.1 Epidemiology of ralstonia pickettii in pediatric clinical settings

In pediatric populations, the presence of this microorganism is strongly linked to hospital environments where moisture, invasive devices and repeated airway manipulation support its persistence. A review that compiled 86 pediatric cases attributed to *Ralstonia* species found that *R. pickettii* accounted for 81.4%, exceeding *R. mannitolilytica* (15.1%) and *R. insidiosa* (3.4%) (2). This pattern is consistent with clinical ecology studies that have documented its presence in hospital water systems, contaminated solutions and respiratory equipment, confirming its preference for humid, nutrient-poor environments (12). Environmental and clinical investigations have reported recovery rates of up to 10% from respiratory samples and hospital devices, reinforcing its ability to persist in healthcare settings (13). Outside the pediatric context, low but recurrent isolation has been reported in blood cultures from units with frequent invasive manipulation, suggesting environmental circulation in complex clinical environments (14). These findings indicate that high-complexity pediatric services, particularly those relying on intensive respiratory support, represent the settings where exposure to *Ralstonia* is most likely (15, 16).

Studies in tracheostomized children provide additional insight into its epidemiological behavior. These cohorts show exogenous acquisition in respiratory cultures obtained after the procedure, even when initial samples were negative, demonstrating the presence of active environmental reservoirs in ventilatory equipment exposed to continuous humidity (17). This observation aligns with changes documented in respiratory microbiota during prolonged ventilation, where colonization reflects the microbiological characteristics of the environment rather than the patient's baseline flora (18).

Reports in neonates offer key information on more severe presentations associated with this microorganism. In several episodes of early-onset sepsis, blood culture isolation was linked to exposure to clinical products that were later shown to be contaminated, predominantly affecting premature infants with immunological immaturity and dependence on invasive devices (19, 20). This pattern is consistent with a neonatal outbreak in which five very preterm infants were colonized after endotracheal instillation of contaminated saline; the investigation demonstrated intrinsic contamination in up to 65% of vials, highlighting the organism's ability to withstand manufacturing processes and spread through standardized clinical products (21). Additional evidence from high-humidity environments, such as burn units, shows that *R. pickettii* can appear in open wounds manipulated with moist materials, although at low frequencies, reinforcing its link to hydrated surfaces and materials (22). These scenarios illustrate the difficulty of early reservoir identification in neonatal units, where multiple inputs can function as silent vectors of transmission (23).

In neonatal care, the relationship between infection and invasive devices is a consistent finding. Recent manipulation of catheters, respiratory circuits or infusion systems often precedes clinical deterioration, supporting their role as vectors of transmission (24, 25). This evidence is reinforced by a multicenter pediatric outbreak in which 34 patients, most of them children, acquired *R. pickettii* through contaminated saline used in irrigation and humidification procedures; molecular analysis confirmed clonal identity among cases, establishing this as one of the largest outbreaks associated with the genus (26). The importance of humid devices as reservoirs is further supported by a prematurity-associated outbreak caused by *R. mannitolilytica*, where reused humidifiers allowed bacterial persistence in internal cartridges and tubing, demonstrating that heating and humidification systems can harbor environmental bacilli despite standard cleaning procedures (27). These findings are consistent with cohorts showing that respiratory circuits can function as persistent reservoirs capable of sustaining colonization for weeks (28).

From an environmental perspective, *Ralstonia* species exhibit notable survival in low-nutrient aqueous solutions and can form biofilms that resist standard cleaning procedures, which explains the prolonged duration of outbreaks involving respiratory and humidification equipment used in pediatrics (2). Microbiological studies confirm that these organisms establish dense, highly resistant biofilms on plastic surfaces, water distribution systems and equipment exposed to prolonged moisture, even after repeated disinfection cycles (12). In settings where contaminated reservoirs are integrated into centralized humidification or internal water distribution systems, spread can extend to general hospitalization areas outside critical care (29). Prolonged viability has also been demonstrated under environmental conditions that resemble devices routinely used in children, supporting its potential to sustain nosocomial transmission despite standard cleaning and maintenance protocols (30).

### 3.2 Environmental sources and routes of transmission in clinical settings

The ability of *Ralstonia pickettii* to persist in clinical environments is supported by its affinity for water systems, capacity to form biofilm and genomic features that promote tolerance to disinfectants and stress conditions. Its presence has been documented in water networks, presumed sterile solutions, humidifiers and complex medical devices, facilitated by mechanisms that include chlorhexidine resistance, passage through 0.2 μm filters and a repertoire of environmental adaptation genes (2, 31–33). This stability is reflected in outbreaks linked to intravenous solutions, heparin, irrigation fluids and contaminated production water, where the organism has demonstrated the ability to bypass filtration systems and persist across entire industrial batches (7, 21, 27, 34–37). These characteristics explain why outbreaks associated with contaminated solutions arise both from manufacturing failures and from prolonged accumulation within storage tanks or distribution systems, leading to transmission through irrigation procedures, nebulization or clinical rinsing solutions (38, 39). Detection of the microorganism in moisture-prone points of water networks and on surfaces of extracorporeal devices confirms that residual humidity can act as a persistent reservoir even outside pharmaceutical products (40, 41).

In devices containing internal water circuits, the combination of stagnant water, structural defects and repeated handling promotes surface colonization and dispersion to vascular or respiratory lines. In pediatric ECMO units, genomic concordance between environmental and clinical isolates confirmed this route, illustrating that even equipment subjected to routine checks can act as reservoirs when residual moisture persists (33, 42). Similar outbreaks have been reported in humidification systems and extracorporeal devices, where internal biofilm accumulation enabled recurrent contamination despite standardized cleaning procedures (27, 35, 37, 41). Additional cases have been linked to heparinized syringes and vascular solutions in which intrinsic contamination introduced the organism directly into the bloodstream, even when aseptic technique was appropriate (7, 43).

Respiratory devices represent another important route. In ventilated neonates and tracheostomized children, tubing, humidifiers and valves have been shown to transfer environmental bacteria into the lower airway, particularly in the presence of vulnerable stomas or repeated interventions. The detection of exogenous colonization by *Pseudomonas pickettii*, now recognized as *Ralstonia pickettii*, in patients with no compatible baseline flora confirms that respiratory equipment can serve as a direct bridge between the environment and the host (17, 28, 44). This risk has appeared in outbreaks involving contaminated endotracheal instillation solutions, which introduced the organism into the airway and facilitated spread through ventilatory circuits (26). High-flow systems and humidification equipment have also harbored persistent biofilm, with documented transmission between devices and patients even after disinfection protocols were applied (27, 37, 45). The presence of environmental nonfermenting organisms in intensive care settings and high-humidity environments supports the ease with which these bacteria spread through aerosols or wet surfaces, favoring cross-colonization events (8, 22, 46, 47).

In immunocompromised patients, exposure to contaminated intravenous solutions or irrigation systems can lead to rapid progression to sepsis, particularly when long-term catheters are in place (48). Transmission has also been linked to peritoneal solutions, urinary irrigation systems and previously colonized catheters, where accumulated biofilm allows reinoculation of the bloodstream or mucosal surfaces in vulnerable hosts (49). Environmental introduction can also manifest as clinical colonization without a clear epidemiological link, highlighting the influence of host susceptibility in infection expression (50–52). The identification of *Ralstonia* in the intestinal microbiota of hospitalized patients suggests that the host may become a secondary reservoir, contributing to environmental circulation and complicating infection control (53).

Confirmation of these transmission routes depends on accurate diagnostic tools. MALDI-TOF combined with genomic sequencing allows differentiation of true outbreaks from pseudobreaks, reconstruction of transmission chains and attribution of origin to medical products, water systems, devices or the host, which is essential for pathogens with multiple potential reservoirs (2, 54). These techniques have demonstrated clonal relationships between clinical and environmental isolates across outbreaks and have ruled out erroneous sources through comparative analysis of genomic profiles and pulsed-field patterns, improving accuracy in identifying environmental reservoirs and failures in manufacturing or reprocessing (38). The available evidence indicates that transmission of *R. pickettii* in clinical environments arises from the convergence of water reservoirs, colonized devices and host susceptibility, a scenario that requires ongoing environmental surveillance and integrated control strategies.

### 3.3 Cellular and molecular determinants of pathogenicity

The pathogenicity of *Ralstonia pickettii* results from a coordinated set of cellular and molecular mechanisms that support environmental persistence, adhesion to abiotic surfaces and human epithelium, tissue colonization and activation of host inflammatory pathways.

#### 3.3.1 Cellular adaptations for environmental persistence

*R. pickettii* exhibits a physiology adapted to survival in nutrient-poor aqueous solutions. Its outer membrane structure and metabolic regulation allow tolerance to osmotic and thermal variation, maintaining viability and growth in physiological solutions, distilled water and clinical preparations, which contributes to its persistence in water distribution systems and medical products (4). The bacterium can pass through 0.45 μm and 0.2 μm sterilizing filters, a property linked to its flexible morphology and cell envelope architecture, a phenomenon documented in multiple outbreaks involving intrinsically contaminated solutions (32). Evidence from hemodialysis systems shows persistence within hydraulic circuits, including low-flow points and disinfected internal surfaces, reinforcing its adaptation to oligotrophic environments with variable hydrodynamics (48).

#### 3.3.2 Cellular adaptations for environmental persistence

Biofilm formation is a major determinant of persistence. The bacterium produces an extracellular matrix composed of polysaccharides and structural proteins that enhance adhesion to pipelines, catheters and internal surfaces of clinical equipment, decrease antimicrobial penetration and support long-term survival (2, 4). Biofilm regulation is mediated by homoserine lactone–based quorum sensing, which coordinates the expression of genes involved in biofilm maturation, antimicrobial resistance and population density (32). The combined effect of biofilm and quorum-regulated signaling increases tolerance to biocides, including chlorhexidine, which has been repeatedly demonstrated in hospital water systems (2).

#### 3.3.3 Mechanisms of adhesion, colonization and tissue invasion

Fluorescence *in situ* hybridization and laser microdissection studies show that *Ralstonia* can adhere to intestinal epithelial cells and form microcolonies at the mucosal interface, supporting effective adhesion to human tissue (55). These analyses reveal aggregates embedded in mucosal layers, maintained by outer membrane adhesins and the ability to form compact structures in inflamed tissue. This pattern suggests that surface determinants enable interaction with the mucous layer and resistance to mechanical clearance, promoting persistence and partial evasion of local immune defenses (55).

#### 3.3.4 Molecular determinants: lipid metabolism, LPS biosynthesis and immune modulation

Metabolomic studies identify an expanded set of enzymes involved in host lipid degradation, including phospholipase A1, phospholipase A2, medium-chain acyl-CoA dehydrogenase and long-chain acyl-CoA dehydrogenase. These enzymes participate in the breakdown of complex lipids, production of acyl-CoA and energy generation through beta-oxidation (56). They support near-complete consumption of oleate and linoleate within 24 to 48 h in culture, altering the availability of essential fatty acids and promoting a proinflammatory microenvironment.

The bacterium also contributes to lipopolysaccharide biosynthesis, particularly lipid A, which drives Toll-like receptor activation and systemic inflammatory signaling (56). These molecular features align with reviews describing low-permeability outer mem-branes and active efflux pumps across the genus, which increase resistance to antibiotics and biocides (2). Contribution to amino acid and B-complex vitamin biosynthesis suggests metabolic plasticity that enhances competitiveness in polymicrobial environments and supports long-term persistence in tissues (56).

#### 3.3.5 Polymicrobial interactions and genetic stability

The presence of *R. pickettii* alongside *R. insidiosa* and *R. detusculanense* in inflamed tissue, all sharing more than 99% genetic similarity, indicates clonal stability and functional coexistence within complex ecosystems (55). The organism remains viable in liquid hospital environments without genomic degradation, suggesting limited selective pressure and efficient adaptation to low-substrate conditions (4). Similar stability has been observed in environmental outbreaks, where a single lineage persisted in hydraulic systems despite disinfection cycles and flow variation, supporting its integration into mixed microbial communities and its persistence in low-flow niches (48). These elements form an ecological profile that blends active lipid metabolism, LPS production and biofilm formation, providing competitive advantages for colonization and survival.

#### 3.3.6 Integrative perspective

The cellular and molecular determinants of *R. pickettii* define a pathogenic profile built around biofilm formation, quorum-mediated communication, epithelial adhesion, lipid-degrading enzymes, lipid A biosynthesis, efflux pump activity, low-permeability membranes and metabolic adaptations that support survival in both water systems and human tissues. Environmental evidence reinforces this perspective, as the species has persisted in hospital hydraulic systems exposed to disinfection and variable flow conditions, confirming its ability to maintain stable microcolonies and withstand standard control measures. The integration of experimental, tissue-based and environmental findings indicates that this organism has developed efficient strategies to resist sterilization, colonize medical devices, persist in aqueous systems and modulate host inflammatory responses, explaining its clinical impact in hospital settings and its role in difficult-to-eradicate infections.

### 3.4 Clinical manifestations across pediatric age groups

Clinical manifestations of infections caused by *Ralstonia* species vary by age, immune status and mode of acquisition. Reported cases range from critically ill neonates to adolescents with severe central nervous system involvement, producing a broad clinical spectrum summarized in Table 1.

**Table 1:** Pediatric clinical patterns associated with Ralstonia species.

Clinical pattern	Pediatric age groud	Features
Persistent fever	Infants, oncology patients, postoperative children	May be the only early sign; useful for early suspicion
Device-associated disease	Neonates, PICC, shunt, EVD	Central element in pathophysiology; strong association with biofilm
Asymptomatic colonization	Neonatal and pediatric ICUs	Essential for differentiating colonization from true infection
Respiratory involvement	Neonates and preschool children	High risk of progression in immunocompromised hosts
Neurological disease	Preschool children, school-aged children, adolescents	High severity; requires CSF analysis or neuroimaging
Variable laboratory findings	All groups	No uniform pattern; depends on focus and host factors
Improvement after catheter removal	Neonates, oncology patients, postoperative children	Key distinguishing feature compared with other pathogens
Pattern	Populations	Key features

Table 1 presents the predominant clinical patterns of pediatric *Ralstonia* infections, highlighting age groups and key features. Abbreviations used are listed in the Abbreviations section before the Annex.

#### 3.4.1 Neonates

Neonates represent the most vulnerable group, with early or late-onset sepsis linked to catheter use and severe respiratory compromise. Fulminant cases in extremely premature infants often begin with nonspecific signs such as apnea, bradycardia, lethargy and progressive respiratory distress, followed by rapid hemodynamic deterioration and, in some instances, disseminated intravascular coagulation and cardiovascular collapse (14, 57–59).

Milder presentations include fever, tachypnea, abdominal dis-tension and variable increases in inflammatory markers (59–61). Respiratory involvement is frequent, especially in intubated neonates, where lower-airway colonization may remain asymptomatic in the setting of severe underlying disease (47, 62). True infection may lead to pneumothorax, progressive infiltrates and escalating ventilatory requirements (25, 61). The association with invasive devices is consistent, and clinical improvement often follows removal of umbilical or central catheters (60, 63, 64).

#### 3.4.2 Infants

In infants, presentation tends to reflect underlying comorbidities. Urinary infection due to *R. mannitolilytica* may manifest as persistent fever, dark urine, vomiting, oliguria and failure to thrive, as seen in an infant with congenital urinary obstruction and repeatedly positive cultures (65). In immunocompromised infants or those with indwelling catheters, recurrent fever with elevated C-reactive protein and bacteremia are common and often resolve only after catheter removal (14, 63, 65).

#### 3.4.3 Preschool children (1 to 5 years)

Preschool children show more heterogeneous presentations, including cardiovascular, respiratory and systemic involvement. A case of endocarditis due to *R. pickettii* presented with prolonged fever, headache, vomiting and pallor, followed by development of a new murmur and rupture of a mycotic aneurysm with intracerebral hemorrhage (24). Atypical pneumonias have also been reported, with persistent fever, productive cough, crackles and radiologic progression to cavitary consolidation that required diagnostic bronchoscopy (8, 66, 67). In children with cancer or immunosuppression, febrile neutropenia predominates, with persistent positive blood cultures that clear after catheter removal (8, 63, 64, 68, 69).

#### 3.4.4 School-aged children (6 to 12 years)

In school-aged children, infections are more frequently localized and occur in immunocompromised or postoperative hosts. Postoperative meningitis has been reported, presenting with persistent fever, cerebrospinal fluid pleocytosis, headache and positive cultures from an external ventricular drain (70).

Severe respiratory infections also occur in children with immune disorders, including a case in a patient with chronic granulomatous disease who developed fever, respiratory compromise, pleural effusion and granulomatous pulmonary lesions (8, 39, 71). Persistent airway colonization without clinical illness is possible in critically ill school-aged children, emphasizing the need to distinguish colonization from true infection (47, 62).

#### 3.4.5 Adolescents

Adolescents may present with patterns typical of younger pediatric groups as well as features resembling early adult disease. A case of shunt infection involved high fever, tachycardia, abdominal pain, peritoneal collection and meningoencephalitis, with fever persisting until shunt replacement (70). Neurological manifestations may result from hematogenous spread from contaminated devices, underscoring the importance of strict management of access sites and ventricular shunts in this group.

### 3.5 Diagnostic limitations and common pitfalls in laboratory identification

Identification of *Ralstonia* species in pediatric settings remains difficult because of their close phenotypic similarity to other non-fermenting Gram-negative bacilli and the limited resolution of routine diagnostic systems. Conventional platforms such as API 20NE, RapID NF Plus and VITEK 2 often produce inconsistent results that classify isolates as *Alcaligenes, Comamonas, Pseudomonas* or unrelated organisms. These errors become more frequent when growth is slow or scant, which reinforces early assumptions of contamination and delays microbiological confirmation (25, 27, 34, 72–74).

Similar limitations occur in respiratory samples from children, particularly in cystic fibrosis, where complex microbiota and heterogeneous tissue compromise culture yield and lead to discrepancies with molecular testing. *Ralstonia* may be detected by sequencing without growing in culture because of low biomass, overgrowth by dominant flora or irregular distribution within lung tissue (16, 29, 61).

Reduced biochemical profiles and the use of limited identification panels further contribute to misclassification, as documented in outbreaks where isolates were misidentified due to incomplete sugar panels or restricted discriminatory capacity of automated systems (38, 46, 50). Taxonomic proximity to the *Burkholderia cepacia complex, Pandoraea, Achromobacter* and *Stenotrophomonas* explains the frequent misidentification of *R. pickettii, R. mannitolilytica* and *R. insidiosa*. These species share overlapping biochemical profiles and similar behavior on conventional and selective media, including growth on agar formulated for BCC or failure to grow on media intended for these species. This produces both false positives and false negatives and complicates interpretation in children with chronic respiratory disease (51, 75–77).

Historical nomenclature such as *Burkholderia pickettii* adds further ambiguity in blood cultures and catheter samples, making it harder to distinguish infection, colonization and contamination and contributing to diagnostic delays in multiple pediatric outbreaks (15, 23, 52, 78, 79). Surveillance studies have shown that *Ralstonia* can coexist with other structurally similar nonfermenters, which leads to misassignment in laboratories with outdated databases (80).

Molecular methods improve diagnostic precision but have limitations when used alone. The 16S rRNA gene shows more than 98% identity across *Ralstonia* species, which reduces discriminatory capacity and explains ambiguous reads and culture-sequencing discrepancies in low-biomass samples. Species-specific PCRs may miss recently described species or closely related phylogroups that appear only in broad *Burkholderia-Ralstonia-Pandoraea* assays, creating false negatives in laboratories reliant on narrow panels and necessitating genotypic confirmation (16, 61, 72, 73, 81). This issue has been documented in outbreaks where VITEK 2 misidentified *R. mannitolilytica as R. pickettii*, corrected only after 16S sequencing or updated MALDI-TOF analysis, demonstrating that automated systems can fail at both genus and species levels (45, 50). Heterogeneous antimicrobial susceptibility patterns among related isolates add confusion and limit the utility of phenotypic profiles for inferring clonality (38, 46).

The ability of *Ralstonia* to contaminate solutions, equipment, respiratory devices, sterile-labelled vials and hospital water systems creates pseudobacteremias and false outbreaks that closely resemble true infection. Its capacity to pass through 0.2 µm filters, persist within resistant biofilms and survive at low bacterial loads explains initial negative cultures followed by later positive results that appear clinically meaningful (27, 34, 37, 47, 74). In several events, cultures of unopened vials confirmed intrinsic contamination of the product while patients remained clinically stable, which prolonged uncertainty around the clinical relevance of isolates and led to either unnecessary interventions or delayed identification of the true source (47, 78). Similar findings have been reported in Vapotherm devices, where reappearance of *Ralstonia* after disinfection and without clinical use indicated that biofilm maturity determines cleaning effectiveness and can generate false negative results following reprocessing (37). In individual cases, single positive catheter cultures or isolated blood cultures were dismissed as contamination despite compatible clinical findings, illustrating that low-level isolation remains a persistent source of diagnostic error (14, 45, 49, 52, 66).

The evidence shows that diagnostic accuracy depends on integrating phenotypic and molecular tools with careful clinical evaluation. The absence of standardized procedures for prolonged culture, variable interpretation of selective media, incomplete or outdated laboratory databases, contamination-related outbreaks, and overlapping biochemical patterns challenge the ability of laboratories to distinguish contamination, colonization and true infection. These factors support the need for combined approaches that minimize false positives and false negatives and strengthen surveillance and clinical management in pediatric practice (15, 70, 78, 82).

### 3.6 Antimicrobial susceptibility patterns and therapeutic approaches

Available studies show that *Ralstonia* species display a susceptibility profile marked by intrinsic resistance to many beta lactams and aminoglycosides, variable activity of fluoroquinolones and trimethoprim sulfamethoxazole, and the presence of mechanisms such as chromosomal OXA beta lactamases, efflux pumps and *qnr* genes (83, 84). Descriptive series report consistent resistance to ceftazidime, cefepime, piperacillin tazobactam and aztreonam, with inconsistent carbapenem activity influenced by local antimicrobial pressure (85). In pediatric settings, *Ralstonia* is uncommon but follows patterns similar to other nonfermenters, with in-creasing aztreonam resistance and partial fluoroquinolone activity (86). Detection of ESBL and AmpC in expanded microbiological analyses further limits the usefulness of cephalosporins for empirical therapy (86). These observations parallel reports from settings such as cystic fibrosis and burn units, where multidrug resistant phenotypes predominate (22, 40, 80).

Clinical reports reinforce this heterogeneity. One *R. mannitolilytica* isolate showed extensive resistance to cephalosporins, carbapenems and aminoglycosides, with activity restricted to levofloxacin, trimethoprim sulfamethoxazole and tigecycline (83). In contrast, a case of *R. pickettii* meningitis showed broad susceptibility and a favorable response to ceftriaxone (58). In a neonatal cohort, resistance to imipenem and meropenem was accompanied by preserved activity only for ciprofloxacin (60). Recurrent infections linked to catheters or dialysis systems showed progressive resistance and biofilm formation, contributing to therapeutic failure until device removal (9, 49). These recurrences have also been associated with polyclonal infections, which explain inter isolate variability even within the same patient (45, 66).

Outbreak investigations provide additional insight into susceptibility patterns. In a hemodialysis unit, *R. mannitolilytica* showed uniform resistance to third generation cephalosporins, piperacillin tazobactam and meropenem, attributed to expression of OXA 22 and OXA 60, with variable fluoroquinolone and trimethoprim sulfamethoxazole activity (35). During epidemics linked to contaminated water or solutions, *R. pickettii* displayed heterogeneous susceptibility between biotypes, although regimens such as ceftazidime with ciprofloxacin or combinations including tobramycin were effective when the contaminated source was eliminated (36). Its persistence in aqueous environments and ability to pass through 0.2 µm filters highlight the importance of environmental control alongside targeted therapy (49).

Reported resistance rates include approximately 17% for ciprofloxacin, 26% for trimethoprim sulfamethoxazole, 30 to 40% for third generation cephalosporins, 45% for piperacillin tazobactam, 60% for aztreonam, more than 60% for aminoglycosides and up to 38% for imipenem. These figures reflect a restricted therapeutic spectrum influenced by OXA 60 expression (5). Clinical response does not always correlate with *in vitro* activity, and catheter or source removal is often required for resolution (7).

Despite these limitations, some agents retain value in selected cases, including ciprofloxacin, levofloxacin, trimethoprim sulfa-methoxazole and, in defined situations, tigecycline (60, 83, 84). Favorable ceftriaxone response in a pediatric meningitis case should not be generalized without microbiological support (58). Treatment should always follow the individual susceptibility profile, avoiding empirical use of third generation cephalosporins or carbapenems in children without strong microbiological justification. Accurate diagnosis, reliable susceptibility testing and proper source management, including removal of contaminated devices, form the most effective strategy to improve outcomes in pediatric *Ralstonia* infections.

### 3.7 Insights from pediatric outbreaks and advances in infection prevention

Pediatric outbreaks caused by *Ralstonia* species share a central feature: intrinsic contamination of hospital supplies, particularly distilled water and aqueous chlorhexidine solutions used in neonatal and intensive care units (18, 87). The organism persists in water based environments and forms biofilm within distribution systems and preparation containers, which allows silent trans-mission across multiple units without clear spatial linkage (87). This dynamic explains clusters of positive blood cultures emerging across different wards within short intervals, often without clear clinical signs of sepsis, which complicates early detection and delays institutional response (87).

Local preparation of antiseptic solutions has been identified as a critical vulnerability. In several outbreaks, distilled water processed through contaminated resins and large batch preparation of chlorhexidine facilitated dissemination of the same clonal strain across pediatric areas (87, 88). This risk is heightened in neonatal units, where immature immunity and frequent use of invasive devices create conditions favorable for colonization and infection. Fulminant sepsis in an extremely premature infant illustrates this vulnerability, even when a specific environmental source is not immediately identified (57). Coexistence of asymptomatic colonization and absence of clear clinical criteria in many infants further contributes to underdiagnosis and hinders active surveillance (88).

Outbreak investigations show that combining antimicrobial susceptibility profiles with genotypic tools such as RAPD has been essential to confirm clonal relationships and reconstruct transmission routes. Correlation between molecular profiles and susceptibility patterns linked patient blood cultures directly to contaminated batches of chlorhexidine and distilled water prepared in hospital pharmacy settings (87, 88). This evidence supported a focused response aimed at the true source based on consistent microbiological data.

The most effective interventions included immediate replacement of contaminated solutions, adoption of safer purification methods such as reverse osmosis, use of single dose chlorhexidine presentations, review of pharmacy and nursing procedures and expansion of surveillance in affected units (18, 87, 88). These measures-controlled outbreaks and prevented recurrence, reinforcing the importance of prevention strategies centered on identifying and correcting contamination sources.

In resource-limited settings, Ralstonia pickettii assumes particular epidemiological relevance, as structural conditions favor both its emergence and persistence. Multiple outbreaks have shown that local preparation of solutions, reliance on hospital water systems with irregular control, and limited traceability of medical supplies increase the risk of intrinsic contamination and sustained exposure (10, 87, 88). In these contexts, detection is often delayed, since advanced microbiological identification is not routinely available and early events may be misinterpreted as isolated contamination or transient colonization (4). Consequently, outbreaks may spread silently across critical care units before being recognized, with substantial clinical impact on vulnerable populations, especially pediatric and immunocompromised patients (11, 26). These experiences suggest that, beyond its intrinsically low virulence, R. pickettii behaves as an emerging pathogen in systems with structural limitations, where microbiological surveillance, environmental control, and regulatory response face operational barriers that delay effective containment.

## 4 Discussion

Ralstonia pickettii should be interpreted less as an occasional laboratory curiosity and more as a sentinel organism for system-level vulnerabilities in healthcare delivery. Across the literature, clinically relevant infections and clusters repeatedly converge on common failure points: aqueous solutions labeled as sterile, water-associated reservoirs, and device-mediated exposure in high-dependency settings. This pattern supports the concept of R. pickettii as an emerging pathogen, not because of intrinsic virulence, but because modern care increasingly relies on complex supply chains and water-dependent processes where small breaches can generate geographically dispersed events and delayed recognition (26, 38).

A critical implication is that early isolates cannot be safely dismissed as contamination when epidemiologic signals are present. Multiple investigations demonstrate that genetically related isolates and lot-linked exposure may underlie what initially appears as sporadic positivity. In practice, this argues for a low threshold to treat Ralstonia detections in sterile-site cultures as potential outbreak markers, particularly when patients share indwelling vascular access or have frequent healthcare contact, and to promptly assess shared products or water-linked materials rather than waiting for case counts to rise. From an infection-prevention perspective, response should prioritize rapid cluster detection, isolate submission for higher-resolution typing when feasible, and product traceability review, because containment frequently depends on identifying and discontinuing the common source (4, 26).

The clinical impact of R. pickettii is heterogeneous and strongly shaped by host vulnerability and care context. While many reports emphasize low virulence, severe outcomes have been documented in critically ill populations, underscoring that the organism's apparent benignity is contingent on immune status, device exposure, and the magnitude and duration of inoculum during contamination events (11). Therefore, clinicians should consider R. pickettii clinically meaningful when isolated from blood or other sterile specimens in immunocompromised patients, neonates, or PICU and NICU populations, particularly in the presence of central lines, prolonged hospitalization, or concurrent exposure to saline-based products (11, 38).

Actionable guidance for practice follows directly from these patterns. For clinicians, repeated isolation from sterile sites or concordant isolates across patients should prompt early source evaluation, careful distinction between transient contamination and true infection based on the clinical syndrome, and timely device assessment, including line removal when clinically indicated. For laboratories, inconsistent identification or atypical non-fermenter profiles should trigger confirmatory strategies and proactive communication with infection prevention teams to avoid under-ascertainment and delayed control (4, 38). For infection control and public health, effective mitigation requires coordinated, multi-agency action, particularly when exposure may involve widely distributed commercial products, combining risk communication, product recall processes, and cross-border information sharing to prevent recurrence and protect high-risk pediatric populations (26, 38).

**Figure 1:**
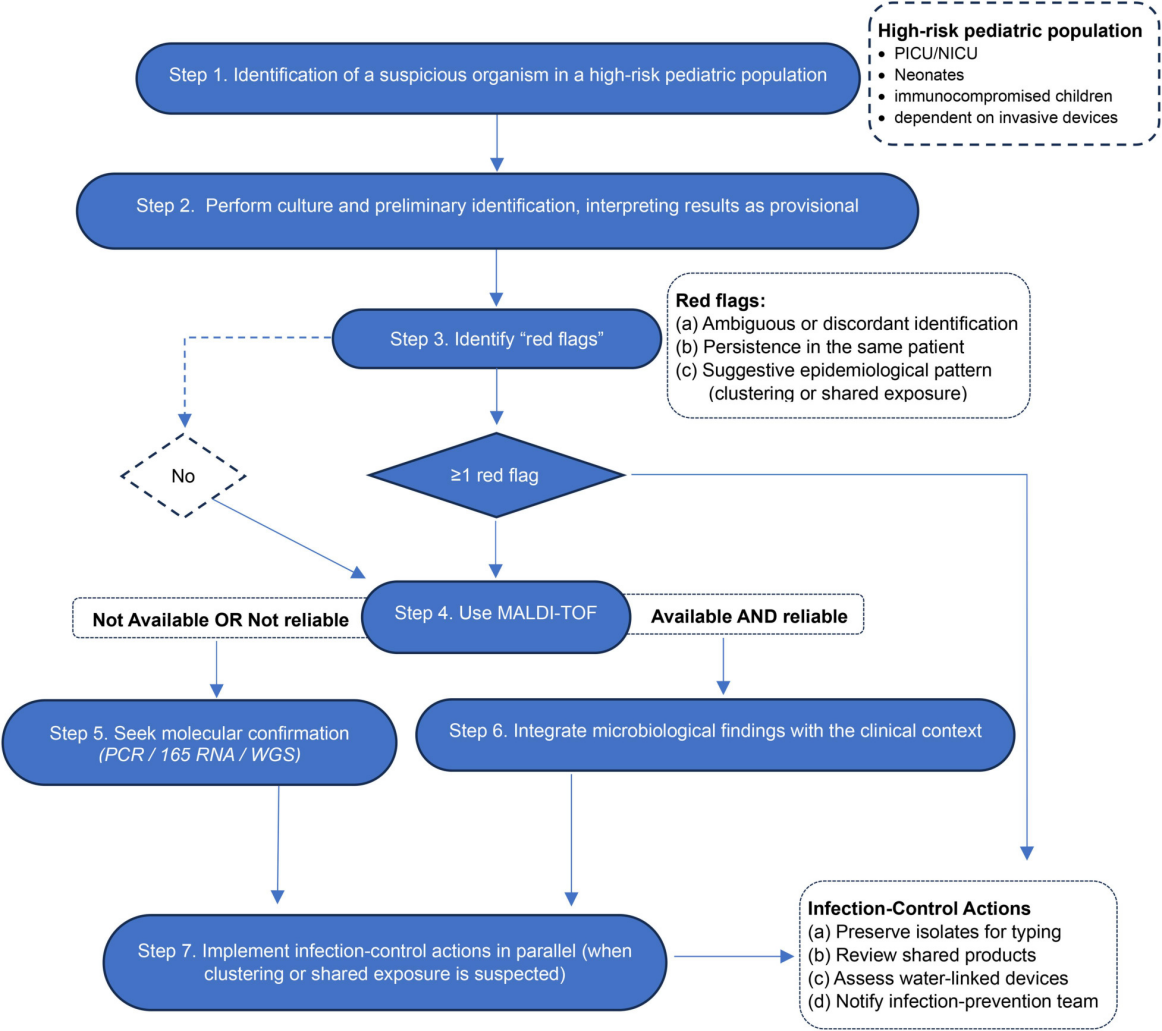
Diagnostic algorithm for suspected Ralstonia pickettii in pediatric settings.

Given the high frequency of misidentification and delayed recognition reported across clinical and microbiological studies, timely diagnosis of Ralstonia pickettii requires a structured, escalation-based approach (Figure 1). Conventional biochemical and automated identification systems are inherently unreliable for Ralstonia spp., owing to substantial phenotypic overlap with other non-fermenting Gram-negative bacilli, particularly the Burkholderia cepacia complex, Pseudomonas spp., and related environmental organisms (12, 13, 73). As a result, isolates are frequently reported as “unidentified non-fermenters” or misclassified, leading to underestimation of clinical relevance and delayed outbreak recognition (38, 76).

Diagnostic suspicion should therefore be triggered whenever a non-fermenting Gram-negative bacillus is recovered from a sterile site in high-risk pediatric settings, such as the PICU or NICU, or when temporally clustered isolates emerge across patients or units (Step 1). Initial phenotypic identification remains part of routine microbiology, yet these results must be interpreted as provisional rather than definitive, given the well-recognized limitations of conventional biochemical systems in this taxonomic group (Step 2). The emergence of red flags, including inconsistent automated identifications, reporting as Burkholderia or “unidentified non-fermenter”, growth on selective media, or the appearance of clustered cases, should prompt immediate diagnostic escalation (Step 3).

MALDI-TOF MS represents a valuable intermediate tool and should be used whenever available, as it enables more rapid genus-level recognition and, with optimized databases, species-level assignment (Step 4). Nevertheless, its performance remains dependent on database completeness and platform-specific libraries, and it may still yield ambiguous or erroneous results in routine clinical laboratories (12, 13, 73). When species-level confidence is not achieved, or when discordant results persist, molecular confirmation using specialized methods becomes necessary, including targeted PCR assays, 16S rRNA sequencing, or whole-genome sequencing (Step 5). Although these approaches provide substantially greater taxonomic resolution, they are not routinely available as point-of-care tests and often require referral to specialized or public health laboratories, inherently introducing diagnostic delay (38, 76).

Final interpretation must integrate microbiological findings with host vulnerability and exposure context in order to distinguish true infection from colonization or contamination (Step 6). Importantly, when clustering is suspected, infection-control actions must proceed in parallel, including isolate preservation for typing, review of shared products, and assessment of water-associated devices and preparation areas, without awaiting definitive molecular speciation (Step 7) (Figure 1). This parallel workflow acknowledges the operational reality that, in most settings, molecular confirmation is not immediately accessible and that timely containment depends on early escalation rather than on definitive taxonomy. A detailed, step-by-step operational description of this algorithm is provided in Supplementary in Annex B.

## 5 Conclusion

*Ralstonia pickettii* exemplifies how contemporary healthcare systems can inadvertently create ecological niches for opportunistic organisms through complex supply chains and water-dependent processes. Its clinical relevance does not stem from intrinsic virulence, but from the convergence of vulnerable hosts, invasive devices, and products presumed sterile. The recurring pattern of delayed recognition and pseudo-sporadic cases across institutions underscores a structural blind spot in routine diagnostics: conventional phenotypic methods are unreliable, while molecular confirmation remains inaccessible in real time for most centers. Treating early isolates as inconsequential contamination perpetuates this gap and allows preventable events to evolve into outbreaks. Recognizing R. pickettii as a sentinel organism reframes each detection as a potential indicator of system-level failure rather than as an isolated laboratory anomaly.

The diagnostic framework proposed here operationalizes this shift. By linking microbiological uncertainty with epidemiological signals and embedding infection-control actions within the diagnostic pathway, it prioritizes timeliness over taxonomic perfection. The algorithm acknowledges real-world constraints, particularly the limited availability of molecular tools, and provides a pragmatic route for escalation that is feasible across diverse resource settings. Its core principle is simple: uncertainty in high-risk contexts warrants action, not reassurance. Adoption of this approach can shorten the interval between first isolate and source control, reduce pseudo-outbreaks, and protect the most vulnerable pediatric populations. More broadly, it offers a transferable model for managing other water-associated and emerging healthcare pathogens in an era where safety increasingly depends on anticipating system vulnerabilities rather than reacting to their consequences.

## Appendix A

**Search strategy** The search strategy identified 230 records. After removing 118 duplicates, 112 unique records remained for screening, forming the dataset used for the narrative synthesis in this review.

**Table A1:** Search terms and query structure across databases.

Database	Terms
PUBMED (*n* = 50)	#1 (“Ralstonia pickettii” OR “Ralstonia species” OR “Ralstonia spp”)
	#2 (“pediatric” OR “pediatrics”)
	#3 (“child” OR “children”)
	#4 (“Infant, Newborn” OR “Newborn Infant” OR “Newborn Infants” OR “Infants, Newborn” OR “neonate” OR “neonates” OR “newborn” OR “newborns”)
	#5 = #1 AND (#2 OR #3 OR #4)
Scopus (*n* = 62)	#1 TITLE-ABS-KEY (“Ralstonia pickettii” OR “Ralstonia species” OR “Ralstonia spp”)
	#2 TITLE-ABS-KEY (“pediatric” OR “pediatrics”)
	#3 TITLE-ABS-KEY (“child” OR “children”)
	#4 TITLE-ABS-KEY (“Infant, Newborn” OR “Newborn Infant” OR “Newborn Infants” OR “Infants, Newborn” OR “neonate” OR “neonates” OR “newborn” OR “newborns”)
	#5 = #1 AND (#2 OR #3 OR #4)
Web of science (*n* = 56)	#1 TS = (“Ralstonia pickettii” OR “Ralstonia species” OR “Ralstonia spp”)
	#2 TS = (“pediatric” OR “pediatrics”)
	#3 TS = (“child” OR “children”)
	#4 TS = (“Infant, Newborn” OR “Newborn Infant” OR “Newborn Infants” OR “Infants, Newborn” OR “neonate” OR “neonates” OR “newborn” OR “newborns”)
	#5 = #1 AND (#2 OR #3 OR #4)
Embase (*n* = 62)	#1 “ralstonia pickettii”/exp OR “ralstonia pickettii”
	#2 “child”
	#3 “pediatrics”
	#4 “newborn”
	#5 = #1 AND (#2 OR #3 OR #4)
