# Supplementary material for: Ralstonia pickettii as an emerging pediatric pathogen: a mini-review of current evidence
Source: Front Pediatr. 2026 Feb 23;14:1763328. doi: 10.3389/fped.2026.1763328 (PMC12968233; doi:10.3389/fped.2026.1763328)
Supplement: Supplementary file 1 [file Supplementaryfile1.docx]

**Annex B. Step-by-Step Diagnostic and Infection-Control Algorithm for Suspected Ralstonia pickettii in Pediatric Settings**

**Step 1. Identification of a suspicious organism in a high-risk pediatric population.**

Isolation of a non-fermenting Gram-negative bacillus from a sterile site (for example, blood, CSF, or catheter-related specimens) in high-risk pediatric patients (PICU/NICU, neonates, immunocompromised children, or those dependent on invasive devices) triggers the algorithm. The algorithm should also be activated when two or more microbiologically similar isolates are detected within a short interval (for example, 7 to 14 days) in the same unit (PICU/NICU) or across epidemiologically linked areas sharing processes or exposures, such as use of the same lot of aqueous products (saline, flushes, respiratory solutions), preparation in the same pharmacy or mixing station, or exposure to water-associated devices (humidifiers, nebulizers, distilled-water systems).

**Paso 2. Perform culture and preliminary identification, interpreting results as provisional.**

Proceed with standard culture and biochemical or automated identification according to routine laboratory practice. However, conventional biochemical identification is unreliable for Ralstonia spp. because of substantial phenotypic overlap with other non-fermenting Gram-negative bacilli and variability across diagnostic platforms. Accordingly, these results should be regarded as provisional, serving as working microbiological hypotheses rather than definitive diagnoses.

**Step 3. Identify “red flags” that determine the diagnostic pathway.**

The presence of signals indicating risk of microbiological misidentification or a system-level event (pseudo-outbreak) must be assessed systematically. These “red flags” determine whether the case continues along the standard diagnostic pathway or whether the evaluation must be expanded.

Red flags are present when any of the following occur:

(a) Ambiguous or discordant identification: the isolate is reported as Burkholderia spp., Burkholderia cepacia complex (BCC), Pseudomonas spp., an “unidentified non-fermenter,” or shows discordant results across platforms, suggesting potential taxonomic misclassification.

(b) Persistence in the same patient: repeated isolation from sterile sites occurs in the same patient despite appropriate sampling and clinical management, making a single-point contamination unlikely.

(c) Suggestive epidemiological pattern: patient clustering is observed, with multiple isolates linked in time or space (for example, within 7 to 14 days), or a shared exposure pathway is identified, such as central lines, flush solutions, respiratory products, common preparation areas, or water-associated devices.

If at least one red flag is identified, the finding should no longer be managed as a routine isolate but treated as an event of clinical and organizational relevance. In this scenario, the microbiological and epidemiological evaluation is expanded, parallel activation of clinical review and infection-prevention teams is initiated, and the algorithm advances to the confirmatory steps (Step 4 and, if necessary, Step 5).

If no red flags are identified, the isolate may initially proceed through the standard diagnostic pathway with routine clinical and microbiological surveillance. However, the identification remains provisional, and the subsequent appearance of additional isolates, methodological discrepancies, or changes in clinical or epidemiological context mandates re-entry into the algorithm at this same step. In this way, the process remains dynamic and responsive to temporal evolution.

**Step 4. Use MALDI-TOF MS as a rapid confirmatory tool when available.**

When MALDI-TOF MS is accessible, testing should be performed early as part of the expanded evaluation. This technology provides faster and generally more accurate identification than conventional biochemical methods; however, results must be interpreted in light of database completeness and updating, recognizing that performance varies across platforms and reference libraries. Confidence thresholds established by each system should be strictly respected, and results must be correlated with the clinical and epidemiological context. Subsequent management depends on the MALDI-TOF MS result:

- If MALDI-TOF MS is available and provides reliable species-level identification, defined as a score within the system’s validated range for species assignment and concordant with the type of specimen, the patient’s clinical condition, and the epidemiological setting, the algorithm proceeds to Step 6 (clinical interpretation and management).
- If these criteria are not met, whether because identification is limited to the genus level, the score is low, the result is ambiguous, or there is discordance with prior findings or with the clinical or epidemiological scenario, the algorithm advances to Step 5 for molecular confirmation using reference methods.
- If MALDI-TOF MS is not available, the algorithm should proceed directly to Step 5, as reliable exclusion of misidentification is not possible using conventional phenotypic methods alone.

In this framework, MALDI-TOF MS functions as a rapid triage tool when accessible, while its absence or insufficient resolution systematically directs the diagnostic pathway toward molecular confirmation.

**Step 5. Seek molecular confirmation using specialized laboratory techniques.**
When identification remains uncertain after phenotypic evaluation and MALDI-TOF MS, or when case clustering is suspected, confirmation should rely on specialized molecular techniques that serve as the confirmatory standard. These tools provide higher taxonomic resolution but are not part of routine point-of-care diagnostics in many hospitals and often require an expanded diagnostic pathway or referral to reference laboratories.

Specialized laboratory techniques options include:

(a) Targeted PCR assays, when locally validated and available, useful for discriminating Ralstonia from other clinically relevant non-fermenters;

(b) 16S rRNA gene sequencing, acknowledging that while it enables genus-level confirmation, species-level discrimination among Ralstonia may be limited;

(c) Whole-genome sequencing, particularly indicated when a potential outbreak is being investigated, as it allows assessment of clonal relatedness among isolates and attribution of common sources.

When in-house molecular capacity is unavailable, isolates should be referred to a reference laboratory for definitive characterization.

**Paso 6. Integrate microbiological findings with the clinical context to classify the event.**

Interpretation of the isolate should integrate microbiological data with the clinical and epidemiological context in order to classify the finding as true infection, colonization, or contamination. This decision should be based on the clinical syndrome, the presence of systemic inflammatory response, biomarker profiles, the sterile-site origin of the specimen, and the reproducibility of the isolate in serial cultures.

In high-risk pediatric patients, particularly those with central lines, invasive support, or immunosuppression, isolation from a sterile site should be regarded as clinically significant by default, unless there is compelling evidence supporting an extrinsic origin or sample contamination. In these settings, assuming clinical irrelevance carries a substantial risk of underdiagnosis.

Conversely, repeated detection of microbiologically similar isolates across different patients with minimal or absent systemic symptoms should raise suspicion of pseudo-bacteremia or a pseudo-outbreak, especially when a shared exposure pathway is present. This pattern is characteristic of events linked to common products, aqueous solutions, or water-associated reservoirs and requires an organizational interpretation of the phenomenon rather than one focused solely on the individual patient.

**Step 7. Implement infection-control actions in parallel when clustering or shared exposure is suspected.**

Containment measures should not be delayed while awaiting definitive microbial identification. When clusters are detected, microbiologically similar isolates recur, or there is evidence of shared exposure, a preventive approach aimed at limiting spread and identifying a common source should be activated immediately.

In this scenario, the following actions are recommended in parallel:

(a) Systematic preservation of isolates for typing and comparative analyses;

(b) Review and traceability of shared aqueous products, including saline solutions, intravenous flushes, products labeled as “sterile,” and respiratory solutions;

(c) Assessment of water-associated devices and preparation areas, such as mixing stations, hospital pharmacies, humidifiers, nebulizers, and distilled-water systems;

(d) Early notification of infection-prevention teams and, when the scope of the event warrants it, external public health authorities, in order to facilitate a coordinated and timely response.
